# Tailoring a Skills-Based Serostatus Disclosure Intervention for Transgender Women in South Africa: Protocol for a Usability and Feasibility Study

**DOI:** 10.2196/52121

**Published:** 2025-03-26

**Authors:** Joseph Daniels, Leonashia Leigh-Ann van der Merwe, Sarah Portle, Cikizwa Bongo, Shiv Nadkarni, Remco Petrus Peters

**Affiliations:** 1 Edson College of Nursing and Health Innovation Arizona State University Phoenix, AZ United States; 2 Social Health and Empowerment Feminist Collective of Transgender Women of Africa East London South Africa; 3 Foundation for Professional Development East London South Africa; 4 David Geffen School of Medicine University of California Los Angeles Los Angeles, CA United States

**Keywords:** transgender women, intervention development, relationships, HIV treatment, South Africa, mobile phones, smartphones, skills-based, serostatus disclosure, HIV, HIV prevention, transgender, treatment outcomes, transmission, HIV-discordant partnerships, behavioral intervention, safe sex, human-centered design, viral suppression, Speaking Out and Allying Relationships, LGBTQ2S, LGBTQ, 2SILGBTQ

## Abstract

**Background:**

Transgender women have few interventions to support their HIV prevention and treatment outcomes in South Africa. Further, increased focus should be on intervention development that will reduce HIV transmission within HIV-discordant partnerships, especially for transgender women who navigate gender, sexuality, and relationship stigma. The Speaking Out and Allying Relationships (SOAR) intervention has been developed for sexual minority men to address these outcomes in South Africa. It is a behavioral intervention that is delivered in groups via videoconference to develop coping skills to manage HIV-related stress, assist with disclosure to partners, and establish and maintain safer sex practices with partners. Tailoring SOAR may be feasible for transgender women to support their HIV care while reducing transmission within their relationships.

**Objective:**

This study aims to (1) adapt SOAR for transgender women and test its usability, then (2) assess its feasibility.

**Methods:**

To achieve aim 1, we will use a human-centered design approach to tailor the existing SOAR intervention for transgender women. Interviews and a survey will be administered to transgender women (N=15) to assess intervention preferences. Findings will be used to tailor content like roleplays, scenarios, and media to align with transgender women’s lived experiences navigating HIV and relationships. Afterward, we will conduct a usability test with 7 (47%) of the 15 participants to determine intervention understanding and satisfaction. Participants will be transgender women living with HIV and in a relationship with a man who has unknown HIV status or is HIV-negative. All participants will be recruited using community-based approaches. In aim 2, we will examine SOAR feasibility using a 1-arm pilot study. Transgender women (N=20) will be recruited using aim 1 methods and eligibility criteria, with participants completing feasibility surveys and interviews, as well as behavioral and biomedical assessments.

**Results:**

Intervention adaptation began in May 2023 with interviews. Feasibility pilot testing was conducted with 14 transgender women, with study completion in January 2025.

**Conclusions:**

Transgender women need more intervention options that engage their relationships since these can present barriers to HIV treatment outcomes like hindering viral suppression in South Africa. Delivering an existing yet tailored intervention via videoconference expands its reach to transgender women and allows them to engage with others and learn new skills in a secure setting like their homes. SOAR has the potential to improve relationship dynamics and reduce violence, which will in turn enhance HIV treatment and prevention engagement.

**International Registered Report Identifier (IRRID):**

PRR1-10.2196/52121

## Introduction

### Background

HIV infections among transgender women are 2.2 higher than gay, bisexual, and other men who have sex with men in sub-Saharan Africa due to syndemics consisting of stigma and discrimination that perpetuate violence and victimization, limiting access to HIV prevention and treatment [1-4]. In South Africa, the HIV incidence for transgender women is 31 cases per 100-person years, with studies showing limited pre-exposure prophylaxis (PrEP) and antiretroviral therapy (ART) access and adherence. Based on other African studies, only an estimated 25% of transgender women are virally suppressed [5-7]. HIV prevalence among transgender women in South Africa mirrors the realities that they share with their peers in other Global South settings, with a 52% mean prevalence documented in 3 South African cities [8]. Transgender women’s health and safety remain compromised in South Africa, inhibiting their ability to engage in HIV services to lower transmission risk and improve their overall wellness, and compounding these health outcomes are poor relationship dynamics.

Being in an unhealthy or imbalanced relationship for transgender women can lower their HIV testing behaviors and increase rates of condomless anal intercourse with partners to create trust and intimacy [9-13]. Fear of partner rejection or loss and intimate partner violence may lead transgender women to limit or halt ART use to conceal their serostatus from partners [14-16]. Further, studies increasingly show that transgender women experience negative outcomes of relationship stigma (when partners fear being romantically associated with someone who is stigmatized), which in turn has hindered their ability to discuss HIV treatment with them [15,17]. Although studies with transgender women in South Africa are limited, in the United States, relationship dynamics for transgender women influence HIV risk, in the same ways they do for cisgender women and sexual minority men [17-21]. Additional factors hindering HIV prevention and treatment discussions that transgender may have with a partner include living with them, drug use, alcohol use, education level, and low self-efficacy to use condoms [18]. In sum, cisheteronormative social structures and power dynamics have been shown to result in increased marginalization, increased susceptibility to intimate partner violence, and increased risk of mental issues among transgender women, all hindering HIV prevention and treatment [22-24].

Further, transgender women are less likely to disclose their HIV status to other transgender women and sexual minority men within their social networks, increasing social isolation [25-27]. Only after being out of care and developing AIDS-related illnesses do transgender women disclose their HIV status to an immediate family member in order to secure support for their treatment, but this disclosure does not extend to partners [25,27]. Given a history of HIV care marginalization and discrimination, transgender women often possess inaccurate knowledge and skills in discussing with partners how PrEP and ART adherence, including “undetectable is untransmissible” (U=U) messaging, can work together to reduce transmission and in turn support relationship health [14-16]. Thus, transgender women have ongoing HIV-related stress without the skills to manage this stress and their disclosure to partners and others for support.

The benefits of social and partner support are significant for transgender women. Studies show that transgender women in committed relationships are less likely to engage in HIV-related high-risk behaviors, with researchers suggesting that relationship stability and emotional support lead to more consistent condom use and improved HIV prevention and treatment overall [28-30]. Specifically, higher relationship satisfaction, trust, and commitment were protective factors against HIV transmission among transgender women [17,31]. Additionally, 1 randomized controlled trial found that a social support group intervention further reinforced relationship-related protective behaviors among transgender women [32]. Therefore, a relationship-focused HIV intervention may support treatment and prevention outcomes for transgender women and their partners while garnering the support of family members earlier in their treatment journey.

To address this gap in interventions for transgender women living with HIV in South Africa, the aim of our study is to tailor our existing intervention, Speaking Out and Allying Relationships (SOAR) intervention, with them and then conduct a 1-arm pilot study to assess intervention feasibility in Eastern Cape, South Africa. Specifically, we will tailor SOAR and assess usability for content understandability and satisfaction (aim 1), and then evaluate its feasibility to include retention and any changes in behavioral and biomedical measures (aim 2). We hypothesize that participants will find SOAR feasible, acceptable, and safe to inform a larger efficacy study.

Our SOAR intervention is based on Healthy Relationships (HR), a 5-session, group-based evidence-based intervention to develop individual HIV disclosure risk assessment and safer sexual behavior skills with partners that have demonstrated efficacy in these outcomes in a range of settings [33,34]. The core elements of SOAR are to: (1) develop coping skills to manage HIV-related stress and sexually risky situations; (2) enhance decision-making skills for HIV disclosure to partners; and (3) establish and maintain safer sex practices with partners. The HR intervention was identified based on our preliminary research with sexual minority men and transgender women in South Africa [35]. Additionally, through this preliminary research, we demonstrated that transgender women are willing to complete HIV-related group work and have an interest in mHealth tools, like videoconference modalities, to learn skills in HIV treatment management and disclosure for support [36,37]. Moreover, harnessing high use rates of smartphones by transgender women for intervention delivery is feasible in South Africa [20,38]. Increased evidence shows that transgender women access web-based social networking sites and use SMS for social networking through mobile and smartphone technologies, creating the potential to tag on ART adherence interventions [39,40]. In our research, we found that HIV-positive sexual minority men and transgender women regularly use videoconferencing to support community networking across rural and urban settings, with 2-hour conversation events focused on HIV, sexuality, and gender [37]. Further, mHealth tools (SMS, chat rooms, and web-based video group interventions) for HIV prevention and treatment in South Africa are part of the national strategic plan [41]. As in the original HR, our SOAR intervention integrates media, scenarios, and roleplays for skill development, and these reflect the experience of South African sexual minority men living with HIV and in relationships. In addition to the standard 5 sessions, we include three 30-minute booster sessions to review action plans over 3 months.

### Theoretical Framework

Social cognitive theory serves as the framework for the intervention, which posits that cognition, behavior, and environment interact and influence health outcomes, like HIV risk reduction, disclosure, prevention, and ART adherence. SOAR aims to enhance coping skills for HIV-related stress, self-regulation of disclosure to include risk assessment, treatment, and prevention, and build self-efficacy in healthy behaviors and communication within relationships. Further, the intervention will provide a platform for participants to strengthen coping skills that will support and empower gender affirmation within relationship dynamics that may be experiencing stigma or risk of violence, or desire for improved relationship communication. Within the social cognitive theory model, the intervention will incorporate components such as HIV risk reduction education, treatment education including U=U, HIV prevention strategies like PrEP, and gender affirmation within the context of HIV. Skill-building activities for HIV communication and disclosure risk assessment will be conducted in a confidential group setting. By helping individuals plan for safe sex and consider HIV communication and disclosure to partners, the intervention aims to support ART adherence, empower participants with a sense of agency, and reduce negative feelings associated with their HIV status and internalized HIV stigma.

Our proposed tailoring of SOAR for transgender women is based on high interest to participate among those who attended community outreach activities for recruitment of sexual minority men as part of the original intervention. If this tailoring shows promise of feasibility, then we will conduct a larger randomized controlled trial to assess efficacy and implementation with local agencies that service sexual and gender minority communities in South Africa.

## Methods

### Study Design Overview

The study will use a 2-fold approach, beginning with intervention tailoring and usability assessment (aim 1) and then a 1-arm pilot study of the intervention (aim 2) to evaluate feasibility. Prior to study initiation, a study coordinator will be hired and trained in study procedures and the SOAR intervention and its delivery approach. The coordinator will be a member of the sexual and gender minority community in Eastern Cape and have a history of HIV program work. Coordinator training will include a review of sexual and gender minority health with a focus on HIV treatment and prevention barriers and facilitators for transgender women, gender-affirming care such as hormone replacement therapy, and relationship dynamics influencing HIV care. Additionally, the coordinator will complete training in good clinical practice and ethics and study procedures including data collection. Finally, the coordinator will be trained in the SOAR intervention which will include both didactic and simulation sessions until competency has been achieved. The study coordinator will conduct all study procedures with mentoring provided by the research team.

### Tailoring SOAR for Transgender Women

In aim 1, we will tailor SOAR by using a human-centered design approach. First, we will assess transgender women’s preferences and confirm their relationship dynamics regarding HIV. Then, we will evaluate the functionality of the intervention in a videoconference format (Zoom, Qumu Corp), and usability by gauging participants’ understanding and satisfaction with the SOAR intervention.

The study will be conducted in Buffalo City Municipality, Eastern Cape, South Africa. In this setting, there is a 46% HIV prevalence among transgender women who live in both urban and rural areas [8]. We will recruit transgender women (N=15) living with HIV and in a relationship for more than a month. Recruitment activities will be conducted by study staff at community events focused on transgender women and sexual and gender minorities. Study staff are members of the sexual and gender minority community and allies, and all completed LGBTQ competency training. Interested participants will be provided study explanation and invited to answer questions in the screening survey. Eligible participants will complete written informed consent in the language of their choosing (eg, isiXhosa, Afrikaans, English). The consent form will outline confidentiality and protections of participant information if they are interested in participating in the study. All participants will receive travel reimbursement and refreshments to complete study procedures.

### Preference Assessment and Integration

To assess transgender women’s preferences for the intervention, we will conduct in-depth interviews (IDIs). These interviews will delve into influences on HIV treatment including support and relationship dynamics, and gather feedback regarding various aspects of the intervention, including format and delivery. A semistructured question guide will ask questions in four domains: (1) HIV prevention and treatment knowledge; (2) relationship scenarios involving HIV disclosure and support for role-play development; (3) videoconference delivery preferences; and (4) positive representation of transgender persons in media (web-based, television, and movies) to show during the sessions. Each interview will be 60 minutes long, conducted in the local language (isiXhosa), transcribed verbatim, and translated for analysis. Findings from the analysis will be used to tailor SOAR to the contextual factors influential in HIV treatment and status disclosure for transgender women and to ensure representation of their empowering voices in the roleplays and videos to support skill building.

### Usability Testing

Usability testing will involve 2 tasks: pretesting and IDIs with participants. We will recruit 7 (50%) racially and relationship-length diverse transgender women participants from step 1 for pretesting. The pretesting phase will span 5 weeks, with 2-hour sessions each week. At week 8, 3 weeks after the last session, a group check-in session will be conducted via Zoom. Participants will receive SMS reminders for each session and the Zoom session link. All sessions will be completed together to foster positive group dynamics and will include a set of group rules for this purpose, and to protect the confidentiality of participants and their contributions during the sessions. The interventionist will monitor participant engagement throughout the pretesting and follow up with any missed sessions. Participants will provide their smartphone numbers for session reminders, self-assessment, action plans, and partner referrals delivered through Research Electronic Data Capture (REDCap; Vanderbilt University). The sessions and group check-in will be video-recorded for analysis, and participants will receive a data plan to facilitate their participation. After the pretesting phase, IDIs will be conducted with racially and relationship-length diverse participants over Zoom. The interviews will cover 6 usability domains for SOAR, including session functionality, timeliness and appropriateness, clarity of content delivery, document clarity and management, incomplete sessions, and technical transitions between sessions. The individual interviews in isiXhosa are expected to last approximately 50 minutes. Audio recordings of the interviews will be transcribed and translated into English for further analysis.

### Data Analysis

To understand SOAR preferences and usability, interview transcripts will be cleaned and confirmed for accuracy before being uploaded into the qualitative data management program, Dedoose, where data will be coded and categorized [42,43]. First, the research team will separately analyze each transcript using predefined codes and open coding, and then discuss coding to confirm alignment and coding distinctions in order to clarify codes [16]. Second, using a thematic approach [44], researchers will analyze the coded transcripts to identify SOAR preferences and usability themes as an iterative process. An additional comparative analysis based on age was conducted to identify any distinctions [44-46]. These discrepancies will be resolved by agreement during research team discussions.

### Evaluating the Feasibility of SOAR for Transgender Women

**Figure 1:**
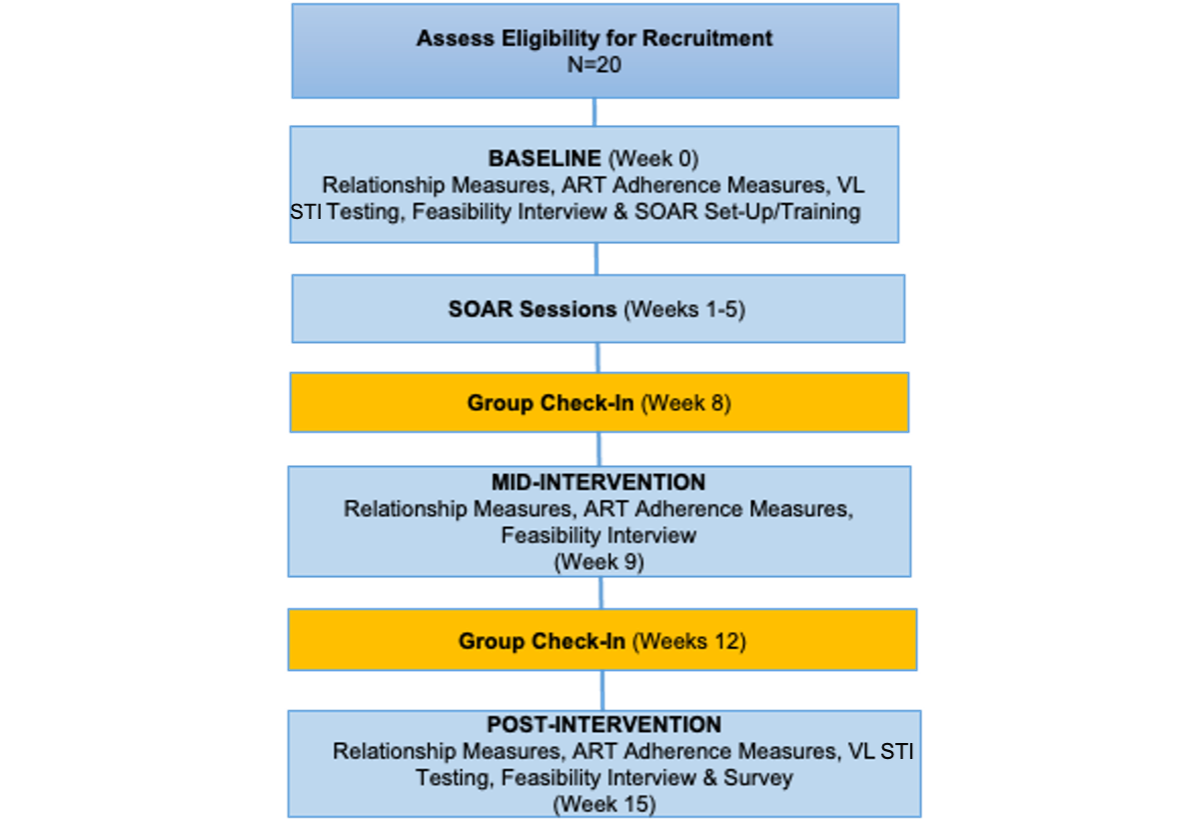
SOAR study design. ART: antiretroviral therapy; SOAR: Speaking Out and Allying Relationships; STI: sexually transmitted infection; VL: HIV viral load.

After SOAR has been tailored, in aim 2, we will conduct a pilot study of the intervention using a 1-arm design to determine feasibility, acceptability, willingness, and safety for transgender women (SOAR study design; Figure 1). Also, we will collect data on ART adherence (viral load) and relationship communication to establish a preliminary understanding of outcomes and implementation of measures in a larger study.

We will recruit transgender women (N=20) living with HIV and suboptimally adherent to ART (missed at 2 doses over 2 weeks), to complete the intervention. Additionally, participants must have been in a romantic relationship with a man for more than 1 month and live in Eastern Cape. For screening, participants will complete a rapid HIV viral load test to confirm HIV status.

There will be 5 participants per group for a total of 4 groups. All participants will be recruited and screened into the study by the trained study coordinator, and all recruitment activities will occur at transgender community–focused events. During the consent process, participants will be provided a general overview of the study and informed about the number of study sessions and expected behavioral and biomedical data collection procedures. As in aim 1, all participants will receive a travel reimbursement and refreshments.

### Procedures

#### Overview

Once enrolled, participants will complete a behavioral survey on REDCap. These measures cover HIV, sexuality, relationship stigma and discrimination, communication in relationships, and HIV prevention and treatment behaviors [47-53]. They will also complete syphilis testing using a rapid diagnostic test (Abbott Determine Syphilis TP), blood draw for HIV viral load, and urine collection for *Chlamydia trachomatis* and *Neisseria gonorrhea* testing. All measures will be administered by study staff.

Afterward, participants will receive training in Zoom, and their smartphones will be assessed and set up for Zoom compatibility. Participants will be notified that they will receive an SMS reminder 12 hours before each session, and then 1 hour before each session, they will receive an SMS with the Zoom link for that session. Also, participants will be informed that during the intervention they will be asked to complete an action plan, and they will receive a partner referral letter via SMS or in paper form (their choice) at the start of the study and can request the letter throughout the study. The referral letter describes the availability of local HIV and mental health services. Each participant will be informed that they will complete 1 SOAR session per week for 5 weeks with 2 check-in sessions afterward.

#### Group Check-Ins

All participants will be invited to attend group check-ins (n=2) via Zoom. The check-in dates and times will be provided during the last session with connection details (eg, Zoom link) sent via SMS to their mobile devices. Participants will be informed that they will receive a reminder about each check-in session 12 hours before and then 1 hour beforehand.

Each session and group check-in will be conducted by the study coordinator and a research assistant who is trained in the intervention*.*

#### Retention Procedures

All participants will be given a card with the study phone number to contact the interventionist if their phone number changes or if they have technical difficulties during the session (the card will not contain information that identifies their participation in the study). The interventionist will phone participants after a missed study visit and SMS each participant monthly to confirm smartphone numbers to support retention.

### Measures and Data Analysis

We will use the same measures as outlined in the parent study to include feasibility, behavioral outcomes for ART adherence and relationship communication, and HIV viral load.

#### Feasibility

The assessment of feasibility encompasses four domains: feasibility [54], acceptability [55], willingness [56], and safety [57]. Feasibility pertains to the ability to successfully recruit and retain participants, as well as facilitate message exchange and engagement in group sessions [54]. Acceptability refers to participants’ preferences and satisfaction with different components of the intervention [55]. Willingness relates to participants’ interest in enrolling in a longer study and their likelihood of recommending the intervention to others [56]. Safety focuses on ensuring the confidentiality and security of participant data and communication both within and outside the intervention.[57]

We will assess feasibility using surveys and interviews. The Self-Intervention Evaluation Form and the Client Satisfaction Questionnaire will be administered to evaluate the acceptability [58,59]. A study-specific Likert scale survey will also be developed to assess feasibility, willingness, and safety [60]. This survey will gauge participants’ perceived ability to exchange messages, participate in group sessions and check-ins (feasibility), their likelihood of enrolling in a longer study or referring other transgender women (willingness), their willingness to provide referral letters to partners, and their perception of the intervention’s confidentiality and security (safety). Also, we will conduct 30-minute interviews with purposively all participants, and these will be conducted at baseline, midintervention, and postintervention [61]. Feasibility will be evaluated by examining participants' attitudes toward various aspects of the intervention, such as video-group interactions, referral letters, and their perceived changes in self-management of ART, HIV risk, and HIV disclosure [62]. Acceptability will be assessed by examining participants’ preferences and satisfaction with specific intervention components and the intervention as a whole [62]. Willingness will be measured by assessing participants’ willingness to use the intervention consistently from start to finish, their openness to using the intervention in different contexts, and their likelihood of recommending the intervention to others [62]. Safety will be examined by evaluating participants’ perceived levels of discomfort with different intervention components and their perceptions of personal safety and the risk of unwanted disclosures [57].

#### Relationship, ART Adherence, and Biomedical Measures

In this study, we will assess the feasibility of collecting behavioral and biomedical measures and provide a preliminary understanding of outcomes. The SOAR intervention is designed to provide transgender women skills in assessing disclosure and related risks, and association communication skills to discuss HIV with partners. We will administer surveys for relationship satisfaction [63] and communication [64], HIV disclosure [65], and HIV treatment adherence [66] Also, we will assess HIV viral load using whole blood. All measures will be administered at baseline through follow-up.

All data will be analyzed using similar procedures as outlined in aim 1 of this study.

#### Participants

For both study aims, all transgender women will be over the age of 18 years, be in a relationship for more than a month, own a smartphone, be comfortable with group discussions about HIV, and be HIV-positive with confirmatory testing using OraQuick (OraSure Technologies) during screening. We will enroll with racial diversity to ensure the representation of multiple voices. Our relationship criteria require participants to have a self-reported romantic or emotionally connected partnership with another individual of any sex or gender, and partners must be either HIV-negative or of unknown status. Additionally, all participants need to live in Eastern Cape and suboptimally adhere to their HIV treatment [66].

### Ethical Considerations

For this study, aim 1 procedures have been reviewed and approved by the University of Cape Town Review Board (FWA00001938) with reliance on the institutional review board at Arizona State University (STUDY00014539). For aim 2, the study procedures were reviewed and approved by the University of Pretoria Review Board (189/2022) with reliance on Arizona State University (16397). All participants completed written informed consent. All participants will complete written informed consent and will receive R150 (around US $10) as travel reimbursement for completing the study activities and an R150 (around US $10) data plan to support their session attendance. All data will be anonymized when reported.

## Results

Staff hiring and intervention tailoring began in May 2023 and were completed in November 2023. During this time, interviews were completed and we identified preferences for the integration of gender-affirming care and South African transgender women’s voices (eg, videos) into SOAR sessions. Feasibility pilot testing started in January 2024 and was completed in January 2025. At this time, there are 14 participants who have completed 1-5 SOAR sessions. Primary feasibility results will be used to inform further adaptation and a clinical trial.

## Discussion

### Expected Findings

The SOAR intervention for transgender women is responsive to South African clinical guidelines for HIV care and addresses barriers to HIV disclosure management and treatment with partners [67]. Specifically, recent clinical guidelines outline a recommendation to deploy mHealth tools for treatment support, as these may allow transgender women to navigate perceived and enacted stigma and discrimination more easily when seeking clinic care [15,16]. Given the established smartphone use in South Africa, and among transgender women, harnessing these tools in HIV interventions is feasible [68]. However, few mHealth HIV interventions have been designed and tested for transgender women [4].

SOAR leverages videoconferencing and group format for delivery. Both modes of delivery have been shown to be highly effective in other settings [69,70], and more research is needed to determine its feasibility with transgender women in South Africa. Further, SOAR has the potential to improve skill-building for HIV status disclosure management that may in turn garner needed support from partners and improve HIV treatment adherence. If partner disclosure and support are not feasible, then SOAR provides participants with coping skills to manage HIV treatment and identification of other support systems for care.

Given that transgender women have some of the highest HIV incidence rates in South Africa and globally [4], it is imperative that we develop more intervention options based on their lived experiences. Integrating mHealth tools into these interventions can empower participants, as it allows them to control engagement based on their safety and needs. If SOAR is feasible as videoconference delivery for transgender women, then we will propose an efficacy study of the intervention to understand the impact on HIV viral load and quality of life. The intervention has the potential to build relationship skills around HIV treatment for transgender women and their partners who are consistently left behind in the HIV response.

### Limitations

The one potential study limitation is smartphone ownership or access. Specifically, although smartphone use is high in South Africa, device costs may be prohibitive for some participants. To facilitate participation such that we have representation of transgender women across socioeconomic standing, we will provide smartphones for participants to borrow, or they may access a tablet with headphones at a community site to attend the sessions.

## Abbreviations

ART: antiretroviral therapy
HR: Healthy Relationships
IDI: in-depth interview
PrEP: pre-exposure prophylaxis
REDCap: Research Electronic Data Capture
SOAR: Speaking Out and Allying Relationships
